# Early-Stage Gas Identification Using Convolutional Long Short-Term Neural Network with Sensor Array Time Series Data

**DOI:** 10.3390/s21144826

**Published:** 2021-07-15

**Authors:** Kai Zhou, Yixin Liu

**Affiliations:** 1 Department of Mechanical Engineering and Engineering Mechanics, Michigan Technological University, Houghton, MI 49931, USA; kzhou@mtu.edu; 2 Department of Chemical Engineering, Michigan Technological University, Houghton, MI 49931, USA

**Keywords:** gas sensory arrays, early-stage gas identification, classification, convolutional long short-term memory (CLSTM) neural network

## Abstract

Gas identification/classification through pattern recognition techniques based on gas sensor arrays often requires the equilibrium responses or the full traces of time-series data of the sensor array. Leveraging upon the diverse gas sensing kinetics behaviors measured via the sensor array, a computational intelligence- based meta-model is proposed to automatically conduct the feature extraction and subsequent gas identification using time-series data during the transitional phase before reaching equilibrium. The time-series data contains implicit temporal dependency/correlation that is worth being characterized to enhance the gas identification performance and reliability. In this context, a tailored approach so-called convolutional long short-term memory (CLSTM) neural network is developed to perform the identification task incorporating temporal characteristics within time-series data. This novel approach shows the enhanced accuracy and robustness as compared to the baseline models, i.e., multilayer perceptron (MLP) and support vector machine (SVM) through the comprehensive statistical examination. Specifically, the classification accuracy of CLSTM reaches as high as 96%, regardless of the operating condition specified. More importantly, the excellent gas identification performance of CLSTM at early stages of gas exposure indicates its practical significance in future real-time applications. The promise of the proposed method has been clearly illustrated through both the internal and external validations in the systematic case investigation.

## 1. Introduction

Due to the increasingly stringent regulation of pollutants and global focus on energy saving, high-temperature gas sensors are highly desired to control combustion processes and monitor toxic emissions [1]. The combustion of fossil fuels as the dominant energy source for automotive and power industries has been the primary cause of global warming and air pollution. Therefore, in situ real-time monitoring and control of combustion-related gases are a top priority in many industrial applications, which requires the sensors to be operated at a high-temperature environment (800~1000 °C) [2]. Besides the conventional high-temperature oxygen sensors, high-temperature carbon monoxide (CO) and hydrocarbons (HCs) sensors are considered necessary to directly determine the fuel combustion efficiency and catalytic efficiency for direct on-board diagnosis (OBD) purposes. Due to the highly aggressive operating environment, only limited sensors have been reported to be able to detect gases above 600 °C [3,4,5]. Ceramic oxides-based gas sensors have been widely explored for high-temperature applications due to their high thermal stability, simple structures, ease of fabrication and low cost. However, lack of selectivity as a common issue for most of gas sensors at lower temperature gets exacerbated at high temperature due to the extremely high reactivity of lattice oxygen in the sensing materials. In addition, the response time is generally longer than gas sensors at lower temperatures due to the time required to reach lattice oxygen equilibrium instead of surface absorption/desorption at lower temperature. This poses a challenge in early-stage gas identification, which allows one to take appropriate action in a promptly manner.

Chemical sensor arrays with subsequent pattern recognition techniques have attracted increasing attention over the past decades, which have enabled non-selective or partially selective chemical sensors to identify multiple analytes or recognize complex chemical environments [6,7]. They have been widely explored to differentiate different common gases (NO_2_, CO, NH_3_, etc.), volatile organic compounds (VOCs), or classify overall chemical mixtures, such as breath analysis and food assessment [8,9,10,11,12,13,14,15]. The data analyses of sensor arrays in earlier studies have been limited to the principal component analysis (PCA) [16,17,18] plots for clustering visualization, and there was no test with unseen data and no quantitative indicator to assess the sensor array performance, such as predictive accuracy of unseen data. The rapid advancement of computational intelligence technology and increasing computing power provide vital supports to achieve efficient and reliable analyte identification and mixture recognition using sensor arrays. A number of parametric or non-parametric machine learning and other optimization techniques have been adopted to classify types of analytes/environments or quantitatively determine concentrations of analytes in a mixture, such as decision tree (DT), support vector machine (SVM), naive bayes (kernel), *K*-nearest neighbor (KNN), random forest (RF), and artificial neural network (ANN) [10,19,20,21,22,23]. While many studies have demonstrated certain level of success for offline analysis, it remains challenging to deploy the chemical sensor arrays for real-time applications. 

Among the abovementioned studies, the sensor array response patterns at equilibrium are utilized as the signature features for the subsequent machine learning emulation. Such machine learning models trained with equilibrium patterns can hardly be applied in the real-time sensing applications, because different kinetics and response time of each sensor in the array may cause response pattern distortion in the transitional phase, resulting in false prediction. The time-series data of sensor array responses, reflecting the underlying time-varying behavior of each sensor upon the exposure to target analytes/gases, have also been exploited to provide extra differentiative features with valuable sensor kinetics information. As reported in the literature, full traces of time-series data of a sensor array response or a set of features extracted from time-series data are often used in various machine learning models, such as logistic regression, DT, SVM, ensemble model and ANN, etc. [15,24,25,26,27]. These models using time-series data have demonstrated powerful capabilities to discriminate structurally similar analytes or complex chemical environments. However, such methods are also not suitable for real-time predication due to the long time needed for the complete acquisition of the entire response curve and sometimes recovery curve in the application. In addition, these models neglect the temporal correlation/dependency in time-series data, that can be any statistical relationship, whether causal or not. It can be very useful to indicate a predictive relationship of temporal time-series data, which can be exploited for real-time applications. Among the state-of-the-art machine learning models, ANN has been increasingly used in various engineering fields including the gas identification area owing to their excellent architecture flexibility and extensibility [26,28,29,30,31,32,33]. The recurrent neural network (RNN), especially its variants so-called long short-term memory neural network (LSTM) and gated recurrent unit (GRU), is capable of capturing the temporal characteristics of the target system. These types of neural networks have been popularly employed for time-series prediction [34], speech recognition [35], traffic forecasting [36], etc. Leveraging upon its unique feature for learning temporal correlation in real-time data, the challenge of early-stage identification mentioned above for high-temperature applications can be potentially addressed.

This study presents a framework for gas identification based on segmented time-series data of a sensor array towards real-time high-temperature applications, with special focus on early-stage identification before reaching equilibrium. In this study, a time-series dataset of a sensor array consisting of four metal oxide nanofibers-based sensors towards three types of reducing gases (CO, methane, propane) at three different concentrations is utilized for algorithm implementation, which is illustrated as a proof-of-principle system. All sensing materials in the array have been reported earlier for high-temperature applications, which are important for combustion process control and toxic emission monitoring in a wide range of industries [37,38,39,40]. The real-time sensor array data intrinsically are of temporal characteristics, which can be synchronously and easily acquired with sufficient length/size. The data length however is substantial under certain circumstances. Hence, a tool embedded with efficient and powerful feature extraction ability is required to facilitate the identification analysis. Over the past years, the hybrid architecture that combines RNN and convolutional neural network (CNN) has emerged, aiming at capturing the temporal correlation of data and extracting the large number of features from data, e.g., high-resolution image or tensor concurrently. The desired performance of an enhanced model has been validated and reported in some scientific fields [41,42,43,44]. Harnessing CNN and RNN collectively, we develop a convolutional long short-term memory (CLSTM) neural network to facilitate the gas identification utilizing time-series data of the sensor array. It is worth pointing out that accurate early-stage identification generally is challenging because all sensor responses in the array are small initially with similar features towards different gases. From an algorithmic aspect, the developed CLTM neural network is expected to differentiate such insignificant difference of features. 

The rest of the paper is organized as follows. In Section 2, the sensor array fabrication and measurements are briefly described, followed by the interpretation of sensor array dataset experimentally acquired in Section 3. Section 4 outlines the computational framework for the early-stage gas identification built upon CLSTM neural network, in which a series of tasks ranging from problem formulation, data preparation, model configuration, to analysis procedures are introduced. Section 5 conducts the systematic model performance investigation to demonstrate the predictive capability of the framework. Concluding remarks are given in Section 6.

## 2. Experimental Procedure

### 2.1. Sensor Array Fabrication

The sensor array was built upon 4 different types of metal oxides nanofibers (NFs), including CeO_2_ NFs (*p*-type), NiO NFs (*n*-type), Ce–Ni–O NFs (*p*-type, Ce:Ni atom ratio = 1:1), and *p*-La_0.67_Sr_0.33_MnO_3_/*n*-CeO_2_ NFs composite (*p*-type, CeO_2_ wt % = 80%) denoted as L_20_C_80_. La_0.67_Sr_0.33_MnO_3_ (LSMO) NFs, CeO_2_ NFs, NiO NFs, and Ce–Ni–O NFs were fabricated by a facile two-step process, consisting of electrospinning and a subsequent calcination process. The detailed preparation procedure and characterization were reported in our previous studies [37,38,39]. L_20_C_80_ nanocomposite was prepared by physical mixing and sonication of LSMO NFs and CeO_2_ NFs [40]. Each metal oxide NFs-based sensor was fabricated on Al_2_O_3_ ceramic screws, as reported elsewhere [45]. Briefly, two platinum wires, serving as two electrodes, were tied on two close threads of the ceramic screw substrate. Metal oxide nanofibers were first sonicated in ethanol and drop-casted onto the substrate to bridge two Pt electrodes.

### 2.2. Sensor Array Measurements

The sensing performance of as-fabricated devices was evaluated at high temperature of 800 °C. The current of each individual sensor at a fixed 1 V DC bias was continuously measured at 20 Hz by a multi-channel electrochemical workstation upon the exposure to different concentrations of reducing gas (CO, CH_4_, and C_3_H_8_) in a dynamic gas flow system [46]. For harsh environment applications, reducing gases react with oxygen at high temperature (800 °C). Therefore, high purity nitrogen was used as the carrying gas of various reducing gases at different concentrations, and 1% O_2_ (in N_2_) was used as the sensor recovering gas. In a typical sensor testing cycle, each sensor placed in furnace at 800 °C was subject to 1% O_2_ for the first 120 s to stabilize the baseline, and then exposed to a particular concentration of reducing gas for 322 s followed by 1% O_2_ for 120 s to recover the sensor. The same cycle was repeated to obtain the responses to gases at different concentrations.

## 3. Sensor Array Dataset

**Figure 1:**
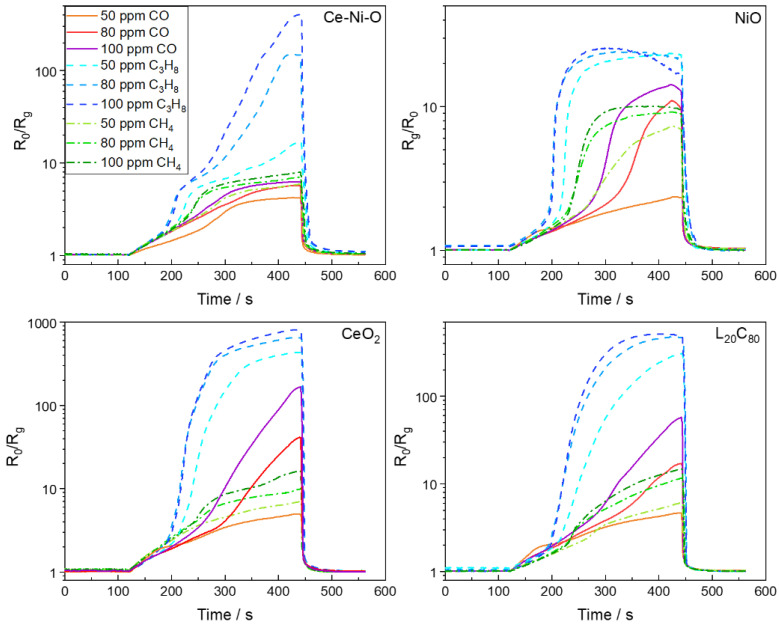
Real-time sensing performance of the sensor array.

The real-time sensing responses of the as-mentioned four metal oxides-based sensors towards 3 reducing gases (CO, CH_4_, and C_3_H_8_) at 3 different concentrations (50 ppm, 80 ppm, 100 ppm) are shown in Figure 1. Among the four sensing materials used in the array, CeO_2_ NFs, Ce–Ni–O NFs and L_20_C_80_ NFs composites exhibited *n*-type behavior, while the NiO NF is a *p*-type semiconductor. The resistance of *n*-type metal oxides decreases upon the exposure to reducing gases with the normalized sensor response defined as *R*_0_/*R_g_*, where *R*_0_ is the initial baseline resistance of the sensor in 1% O_2_, and *R_g_* is the measured real-time resistance in different gases. For *p*-type NiO NFs, the resistance increases in the presence of reducing gases, thus *R_g_*/*R*_0_ was used as the normalized sensor response to keep the value larger than one. For visualization purpose, the logarithmic scale is used in Figure 1 to capture the features in the wide range of normalized sensor responses from less than ten to several hundreds.

As shown in Figure 1, *n*-type CeO_2_ NFs shows very high sensitivity to propane, moderate response to CO and minor response to methane, and *p*-type NiO NFs exhibits relatively low sensitivity to all reducing gases with saturated response to propane at testing levels. Ce–Ni–O NFs was characterized to be NiO nanoparticles decorated on Ce–Ni–O backbone, which significantly suppresses the response to CO and enhances the differentiation capability towards propane at different concentrations [39]. Combining *n*-type CeO_2_ NFs and *p*-type LSMO NFs to form a L_20_C_80_ NFs composite also utilizes the *p*-*n* heterojunction to modulate the selectivity of propane over CO [40]. With improved selectivity towards propane, a single sensor device still cannot differentiate a low concentration of propane versus a high concentration of CO, nor a low concentration of CO versus a high concentration of CH_4_ at an equilibrium state. However, each sensor shows drastically different kinetics profiles (time-varying behavior) towards different gas species, and the time-varying behaviors of these four sensors towards the same gas species are also different, providing signature time-varying features upon the exposure to various reducing gases. Therefore, the time-series data of sensor array response is proposed to be utilized in this study to identify the gas species during the response transitional state. 

**Figure 2:**
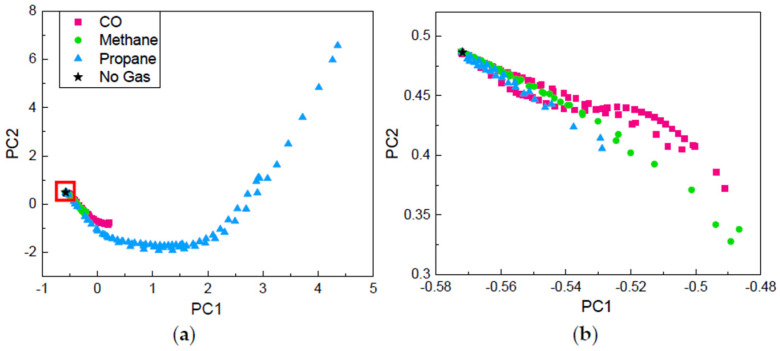
Projections of the segmented time-series dataset onto the two leading principal components for three target gases: (**a**) overall principal component information; (**b**) zoom-in principal component information at initial stage.

It is worth noting that during the first 60 s upon the reducing gas exposure (i.e., 120 s–180 s in Figure 1), all sensors show small normalized responses less than 2, and all real-time sensor response curves during the first 60 s almost overlap with each other, making it very challenging to identify the gas species at early stage. To further assess the difficulty of the problem, we performed principal component analysis (PCA) on the time-series data extracted from the reducing gas exposure region, which starts from 120 s and lasts for 322 s, as shown in Figure 1. Such 322 s time-series data in each test are then segmented into 32 samples/segments, each of which contains 10 s of data. Since there are nine tests, i.e., the sensor responses collected upon the exposure to three types of gases with three concentrations, there are 288 (32 × 3 × 3) gas samples. A “No Gas” sample was added to the sample database to indicate the initial position in the plot of the two leading principal components. The “No Gas” sample also contains 10 s of time-series data with a constant value of 1. Therefore, there are 289 samples in total in the PCA study. The two leading principal components are calculated using SPSS Statistics and plotted in Figure 2. Due to the temporal characteristics of the time-series samples, PCA result shows the certain tendency with respect to time. As can be seen, data points close to “No Gas” (black star) at the leftmost side of the figure are the initial segments upon the exposure to reducing gases, and they gradually move to the right over time approaching to equilibrium. From the figures, it is clearly seen that propane (blue triangle) has the most differentiable features (Figure 2a). However, data points of all three gases are inseparable at the initial stage (Figure 2b), indicating the difficulty of early-stage classification. 

## 4. Gas Identification Framework

In this section, a comprehensive computational framework to conduct the gas identification analysis is presented. The mainstay of the framework, i.e., CLSTM neural network is firstly outlined, followed by the introduction of the problem formulation, experimental data handling, model development and analysis procedures.

### 4.1. Background/Overview of CLSTM Neural Network

Owing to the advancements in computational power and data science, machine learning through neural networks has seen rapid progress in recent years. A neural network essentially is built upon different layers such as input layer, hidden layer, and output layer, by linking the nodes in layers. Hidden layers undertake the direct computation to extract underlying data features. According to the functionality, hidden layers can be further divided into fully connected layers, convolutional layers, and max-pooling layers. While fully connected layers are widely utilized for characterizing the high-level feature correlation, convolutional layers and max-pooling layers are oftentimes integrated into deep learning convolutional neural networks (CNNs) to learn the massive low-level features directly from raw data, such as image and video. 

**Figure 3:**
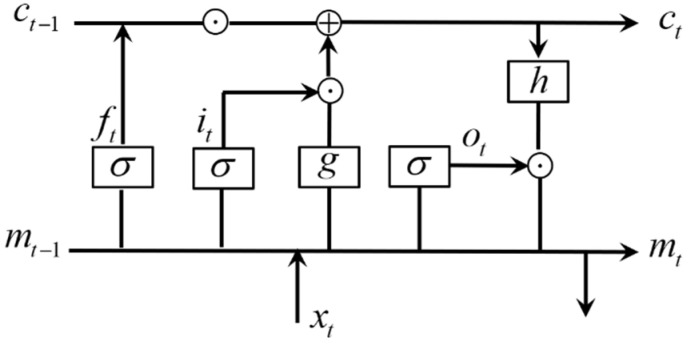
Architecture of LSTM memory cell.

The long short-term memory (LSTM) neural network, as emphasized in the Introduction, has the capability to take the temporal correlation of time-series data into account when performing the learning/training process. The LSTM neural network is a special variant of the recurrent neural network (RNN). The underlying component of the LSTM neural network is the memory cell, which can memorize the temporal state through three different controlling gates, i.e., input, forget and output gates (Figure 3). When the input gate is activated, the input information can be stored into the cell. When the forget gate is activated, the past cell state can be forgotten. The output gate can control if the latest cell output can be propagated to the ultimate state. The mathematical model of the memory cell is described as [41]:

(1) $$f_{t}=\sigma (W_{fx}x_{t}+W_{fm}m_{t-1}+W_{fc}c_{t-1}+b_{f})$$

(2) $$i_{t}=\sigma (W_{ix}x_{t}+W_{im}m_{t-1}+W_{ic}c_{t-1}+b_{i})$$

(3) $$o_{t}=\sigma (W_{ox}x_{t}+W_{om}m_{t-1}+W_{oc}c_{t}+b_{o})$$

(4) $$c_{t}=f_{t}⊙C_{t-1}+i_{t}⊙g(W_{cx}x_{t}+W_{cm}m_{t-1}+b_{c})$$

(5) $$m_{t}=o_{t}⊙h(C_{t})$$

where $f_{t},i_{t},\;$ and $o_{t}$ denote the forget, input and output gates, respectively; $c_{t}$ and $m_{t}$ denote the cell state and cell output; W and b are the weight matrices and bias vectors to be optimized; ⊙ denotes the scalar product; and $x_{t}$ is the input information, which represents the data of *t*-time sequence in one time-series sample. The activation functions used in the cell are expressed as:

(6) $$\sigma (x)=\frac{1}{1+e^{-x}}\;\;\sigma (x)\in [0,1]$$

(7) $$g(x)=\frac{4}{1+e^{-x}}-2\;\;g(x)\in [-2,2]$$

(8) $$h(x)=\frac{2}{1+e^{-x}}-1\;\;h(x)\in [-1,1]$$

Note, σ(x) is a sigmoid activation function, and g(x) and h(x) are the hyperbolic tangent activation functions. If *t*-time sequence is the ending sequence of the time-series sample, the LSTM output can be further derived as:

(9) $$y_{t}=W_{ym}m_{t}+b_{y}$$

The LSTM layer that is comprised of multiple memory cells is a representative type of layer to be incorporated into the LSTM neural network. The CLSTM neural network can be established if the convolutional layers are appropriately integrated into LSTM neural network for extracting low-level features directly from the input. The CLSTM neural network generally is very powerful and efficient in handling the input with the large number of features. It is noteworthy that time-series data usually are lengthy due to the high sampling frequency of digital signal device nowadays, which will be facilitated by the feature extraction via convolutional layers. Therefore, taking advantage of intrinsic features of CLSTM neural network, the challenging gas identification mission proposed in this study can be fulfilled. The particular configuration of the CLSTM neural network architecture will be detailed in the subsequent section (i.e., Section 4.3).

### 4.2. Data Labeling, Time Series Reformatting, and Problem Setup

As indicated in the Introduction, the objective of this study is to achieve the rapid gas identification during response transitional state, which essentially belongs to the classification analysis. Once gas species is determined, its concentration can be identified based on the established sensor calibration curves [46]. To include the concentration impact on gas classification, we create the class labels, each of which points to specific type of gas including three different concentrations (Table 1). Such data fusion/combination increases the feature diversity of each gas class, and hence calls for a powerful classifier with enhanced generalization ability. In addition, for demonstrating an early-stage identification scenario, the testing environment without reducing gases (before the reducing gases are introduced) also is considered as a class, denoted as the “No Gas” class. As discussed in Section 3, the sensors respond slowly to the reducing gas introduction at initial stage, in which not only the sensor response patterns of different gases are similar, but also those response patterns will interfere with that of “No Gas” situation. This indeed poses a challenge in achieving the desired accuracy of early-stage gas identification. 

**Figure 4:**
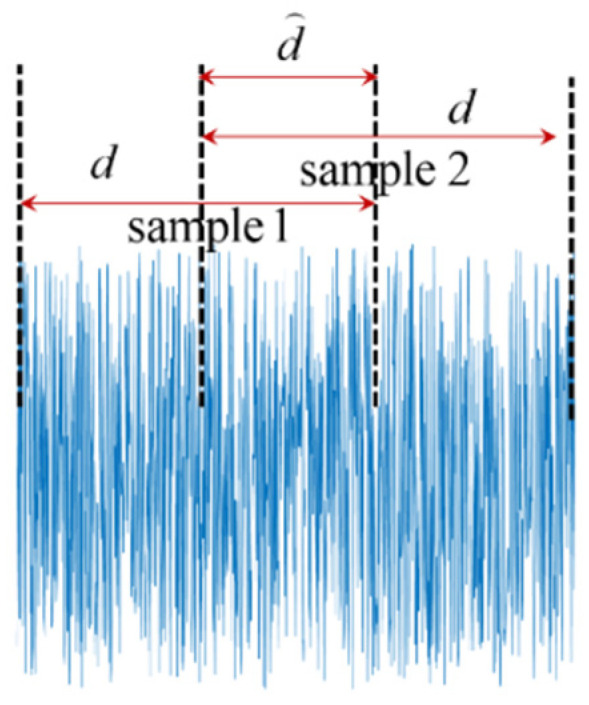
Illustration of time series sliding window with overlapping.

Specific care should be taken to re-format time-series data properly for the ease of CLSTM neural network construction. Each input sample should contain sufficient data points to reflect their underlying features and correlation. However, the traditional data segmentation will yield a relatively small size of input samples. To fully take advantage of the time-series data, we adopt the overlapping sliding-window technique [47] to generate a large number of input samples while the length of each input sample still is sufficient. The idea of this technique is simply illustrated in Figure 4, where the overlapping ratio is defined as $\overset{⌒}{d}/d$. 

**Figure 5:**
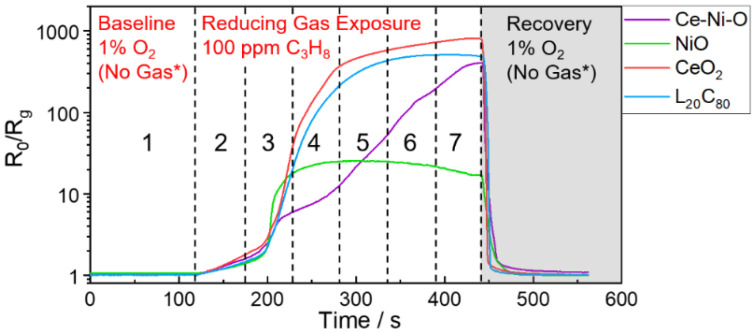
Illustration of time-series data of the sensor array responses to 100 ppm propane as an example, indicating the effective area (grayed-out recovery region is not considered) and the region segmentation (#1–7) for early-stage classification analysis. No Gas* means no reducing gas in the gas flow.

Recall that the experimental sensor sampling frequency is 20 Hz. The whole testing time lasts 562 s, which yields in total 11,241 time points. Since our specific interest lies in the early-stage gas identification, the last 120 s of recovery region is not considered and labeled as an ineffective area, as shown in Figure 5. We specifically focus on the classification analysis of gases within the effective area, including baseline region and reducing gas exposure region, which contain the first 442 s with 8842 time points. The entire effective area/testing time is further segmented into several regions to facilitate the investigation of gas identification accuracy over time. Particularly, the time frame of baseline (“No Gas”) is defined as region 1, and the time frame of gas exposure is uniformly segmented into six regions. As will be shown later, through analyzing the CLSTM model performance upon those local regions, the potential of early-stage gas identification can be clearly illustrated. 

In this study, we select 100 data points for each time-series sample (5 s). With this, we produce all the time-series samples with 0.5 overlapping ratio specified. Different gases are denoted as Classes 1–3. It is worth mentioning that, in order to maintain the class label balance, we trim a few samples of “No Gas” class (Class 4) at the beginning of the testing. According to such set-up, we generate 381 samples for Classes 1–3 and 378 samples for Class 4 (Table 1). The slight size difference is due to the data truncation (division with remainder in this case). Totally, we have 1521 samples for subsequent classification analysis. The general dimension of each input sample is represented with 100×4 (4 is the number of features/sensor responses). CLSTM neural network inherently considers the effect of time lead and delay during the training, which requires each input sample to be further split into different time sequences. Temporal correlation is implicitly characterized by those different time sequences. Here, we specify the number of time sequences for each input sample, i.e., 4, and thus the dimension of each input sample is reshaped as 4×25×4. As compared with other types of neural networks, CLSTM neural network requires more parameters to be specified to prepare the input information with appropriate format for the ease of model training. Those parameters, such as time-series length may play an essential role on the model performance, which is subject to investigation in the subsequent section (i.e., Section 5.1). 

### 4.3. CLSTM Neural Network Architecture Configuration

In this study, each time sequence of each sample is set to have the size of 1×25×4, which can be perfectly fed into the 1-D convolutional layer where 4 is the number of channels. Specifically, 1-D filters in the convolutional layer can directly extract the features of this 1-D vector (single channel) including 25 values. In this context, we develop a convolutional long short-term memory (CLSTM) neural network with the convolutional layers embedded to rapidly and automatically extract the low-level features from the time-series data. The max-pooling layer, as one representative type of pooling layer, is usually added after the convolutional layer. Its purpose is to reduce the dimensions of the resulting feature maps through down-sampling, thereby minimizing the overall training effort of the neural network. Fully connected layer is the layer where its inputs are connected to all units of the next layer. In most neural network models, the last few layers are full connected layers which compile the features extracted by previous layers to establish their correlation with respect to the final output. The LSTM layer that is comprised of multiple memory cells is particularly involved to fulfill the early-stage gas identification purpose. The activation functions such as sigmoid, ReLU, and hyperbolic tangent, etc., are commonly employed after layers to build the nonlinear mapping of input and output, which enhances the capability of network to learn real-world data with nonlinearity. The configuration of CLSTM neural network primarily lies in the layer configuration, which can be implemented following the model training performance. Specifically, during the training process, a small amount of hold-out training data will be left for validation. The training performance can be thoroughly assessed by observing the training and validation loss histories. Underfitting appears to occur when the training loss has the large magnitude order. In comparison, overfitting appears to occur when the validation loss bounces back whereas the training loss continues to decrease during training. The appropriate network architecture is expected to enable the adequate training without underfitting and overfitting, given the available dataset. 

**Figure 6:**
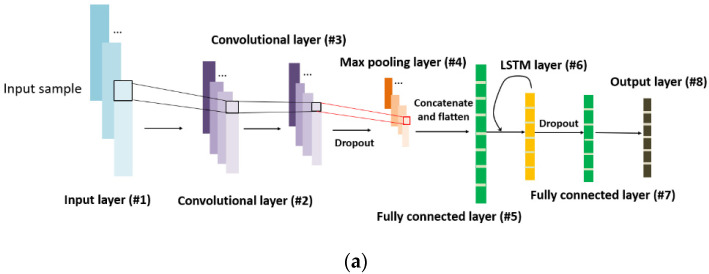

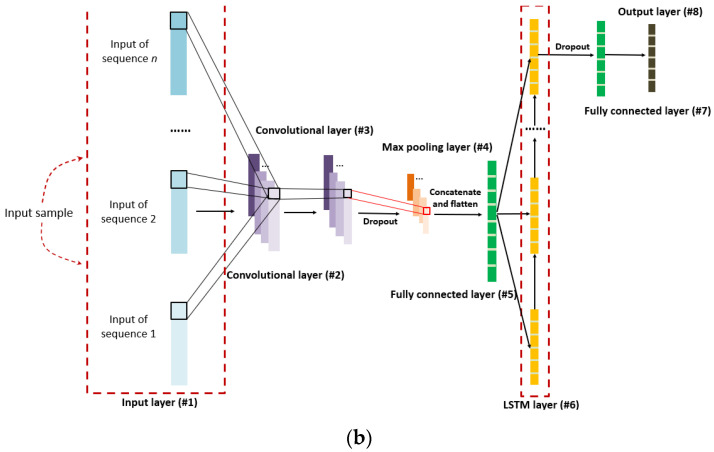
Architecture of CLSTM neural network model (**a**) fold; (**b**) unfold.

In this research, the finalized architecture of the CLSTM neural network is represented in both fold and unfold forms (Figure 6). This model consists of 8 layers, and their properties are detailed in Table 2 for interested readers to reproduce results. It is worth mentioning that the 0.1 dropout is applied into layer #3 and #6 for alleviating the overfitting under small dataset. The stride of filters in convolutional layers is set as 1, and non-zero padding is adopted that yields the diminished output size (Table 2). The input size is determined by the time sequence split of each time series, and output size is identical to the number of gas classes defined earlier.

### 4.4. Baseline Models

To highlight the enhanced performance of the proposed methodology in the subsequent investigation, without loss of generality we utilize some baseline models as a reference, including SVM and multilayer perceptron (MLP), which are well-known and representative machine learning models. They are formulated upon the different fundamental mathematics, which can ensure rigorous performance comparison. Since the development of those baseline models is not the particular focus of this research, only the general background of those models will be briefly outlined below. 

Support vector classifier (SVC) is formally defined by a separating hyperplane that is determined upon so-called “support vectors”. Training the classifier is to maximize the marginal distance between support vectors through optimizing the related model parameters [48]. This method essentially is a kernel-based method, which only requires a specified kernel function, i.e., a similarity function to describe the correlation of data in raw representation [49]. Owing to this unique feature, this method can be operated in a high-dimensional, implicit feature space without computing the coordinates of the data in the space, resulting in a relatively cheap computational cost. Multilayer perceptron (MLP) essentially is a representative type of neural network which only consists of the hidden fully connected layers for data correlation learning [50]. MLP oftentimes is used as an integral element for other complex neural network designs. Because both CLSTM and MLP belong to the neural network family, we also consider MLP as a baseline model. This can fully allow one to observe the notable performance difference caused by the enabled features of the proposed CLSTM neural network model.

## 5. Implementation, Results and Discussions

### 5.1. Performance Investigation of CLSTM Neural Network Model—Comparison with Baseline Models and Examination of Sample Length Effect

Upon the raw data pre-processing and curation procedures defined in Section 4, we implement the gas species classification analysis, and then conduct the systematic performance investigation and comparison. A total of 1522 available data samples are split into 80% training and 20% testing data for model establishment. All baseline models and CLSTM neural network are developed using Python Keras and Tensorflow (Anaconda Spider IDE) [51]. SVM used in this study is built upon the radial basis kernel, in which the related parameters, such as the regularization parameter and variance are treated as hyperparameters. These hyperparameters are firstly discretized into specified grids, and then are determined through the cross validation-based method grid-search upon the available training dataset [52]. A three-layer MLP with each layer including 512, 256 and 128 neurons respectively is established. The total number of trainable parameters in the model is 370,052. The hyperparameters are tuned beforehand in order to achieve the desired model performance. The hyperparameters of MLP and CLSTM neural networks are determined as: epoch size (40), batch size (10), learning rate (0.01) as well as backpropagation optimizer i.e., ‘Adam’ (a stochastic gradient descent optimization algorithm), for unknown weights and biases updating. It is worth mentioning that, as the typical model performance metrics, the underfitting and overfitting are explicitly looked into. All models developed are confirmed without underfitting and overfitting by carefully examining their training and validation losses. For example, for the neural network training, a small set of holdout validation data can be utilized. The training and validation loss curve trends with respect to the epoch can be used to indicate the occurrence of underfitting and overfitting. 

**Figure 7:**
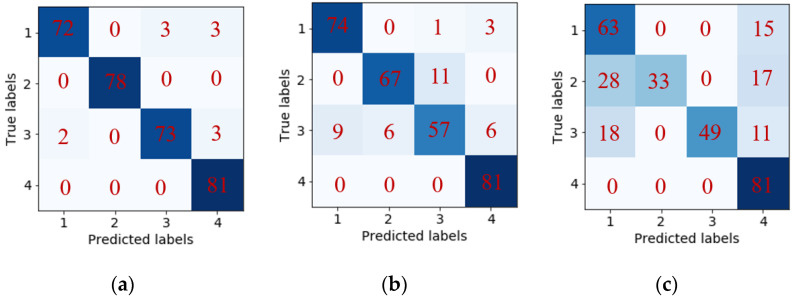
Classification result: confusion matrix (**a**) CLSTM; (**b**) MLP; (**c**) SVM.

**Figure 8:**
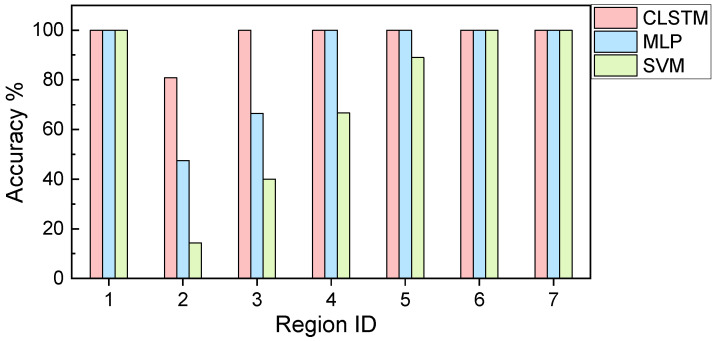
Classification accuracy with respect to the time region (single run).

Once the models are finalized with appropriate hyperparameters, we perform the gas species classification analysis. The classification metric is chosen as “Accuracy” that is the most intuitive measure [53]. It is simply a ratio of correctly predicted observations to the total observations. The classification results, i.e., confusion matrix results of 20% testing samples, under different models are given in Figure 7. Above classification metric, i.e., “Accuracy” is calculated as 96.51%, 88.57% and 71.75% for CLSTM, MLP and SVM, respectively according to the confusion matrix information. This result indicates that CLSTM has the best classification accuracy among these models, whereas SVM has the worst accuracy. Additionally, the classification accuracy of different models over time (i.e., segmented time regions) is shown in Figure 8. The result provides the consistent observation as indicted in Figure 7. Especially, CLSTM outperforms other models over all segmented time regions in terms of classification accuracy. Most of the misclassifications take place at early gas exposure stage (i.e., region 2, in the first 50 s of exposure). However, CLSTM still outperforms other models in terms of classification accuracy. It is noteworthy that almost 100% accuracy of CLSTM can be achieved for the gas identification over the rest of regions.

**Figure 9:**
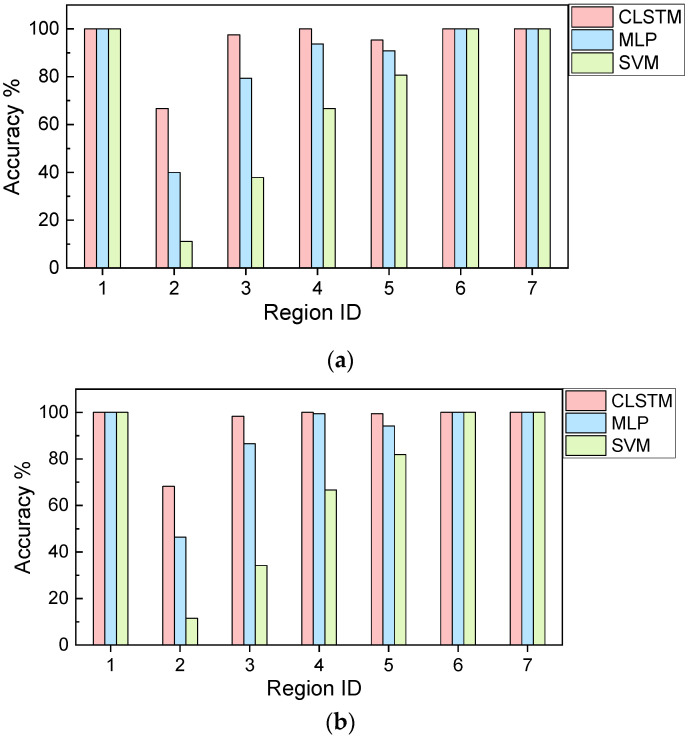
Cross-validation result: classification accuracy of (**a**) 100 data points per sample; (**b**) 40 data points per sample.

While the above results clearly demonstrate the powerful capability of CLSTM for real-time gas identification, it is worth mentioning that the model emulations inevitably are subject to randomness. For example, the training and testing data split is random which affects the model establishment and validation. The neural network model training also has variation due to the random batch generation and stochastic gradient-based parameter optimization even the data split is deterministic. Taking such randomness into account during emulations can lead to the systematic assessment of model performance robustness. In this study, we adopt the repeated random subsampling cross validation to carry out such investigation [52]. Noteworthily, the stratified sampling concept is specifically integrated into the cross validation to enable the label balance for the classification problem [54]. The same training and testing split ratio, i.e., 0.2 (20%) is used and 20 emulations are implemented. The statistical mean of the overall classification accuracy for CLSTM, MLP and SVM is 95.79%, 87.82% and 77.75%, respectively. The associated standard deviation is 1.98%, 4.48% and 3.23%, respectively (Table 3). The accuracy mean trend with respect to the time region is also shown in Figure 9a, clearly illustrating that CLSTM outperforms MLP and SVM, especially with notable performance improvement at early gas introduction stage (i.e., region 2). Moreover, the slightest standard deviation of accuracy in CLSTM shows that CLSTM is insensitive to the randomness, and thus possesses the excellent robustness. As a comparison, MLP yields the most significant standard deviation of accuracy indicating the worst robustness, even its accuracy mean is higher than that of SVM. Overall, a series of results shown above certainly validate the feasibility of CLSTM for fulfilling the gas identification mission in this study.

One may notice that the length of each time-series sample is user-specified, which is worth being investigated. To the best of our understanding, this may affect the MLP and SVM since the time-series length essentially represents the number of features to be dealt simultaneously during model training. For CLSTM, both feature dimension and temporal dependency level that vary with respect to the time-series length may lead to the model performance variation as well. Therefore, in this subsection a different time-series length, i.e., 40 is considered (2 s), and the analysis is performed following the similar procedures shown above. It is noted that only varying time-series length when keeping the same other operating conditions will result in a different total number of samples. In this particular case, 963 samples are produced using 0.5 overlapping ratio-based sliding window for each class. While the feature dimension of each sample is reduced, the significantly increased number of samples generally need more computational cost for training. The cross-validation results, i.e., accuracy mean and standard deviation of 20 emulations in this new case are also appended in Table 3. Comparing the results in Table 3, reducing the time-series length and correspondingly increasing the number of time-series samples improve the overall model classification accuracy slightly. Intuitively, either too large or small length will degrade the model performance, and hence there may exist a best time-series length given particular application context. The accuracy trend obtained (Figure 9b) is similar to that shown in Figure 9a, in which CLSTM has more superior performance than MLP and SVM. As far as the early-stage gas identification is concerned, CLSTM apparently shows good potential/prospect. 

### 5.2. External Validation of CLSTM Neural Network Model

**Figure 10:**
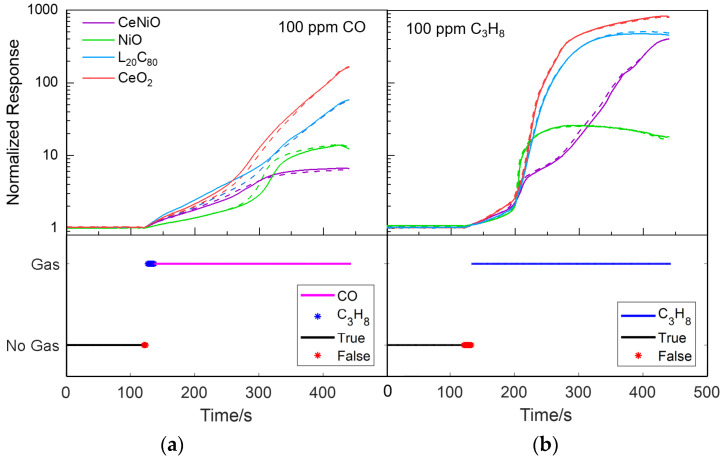

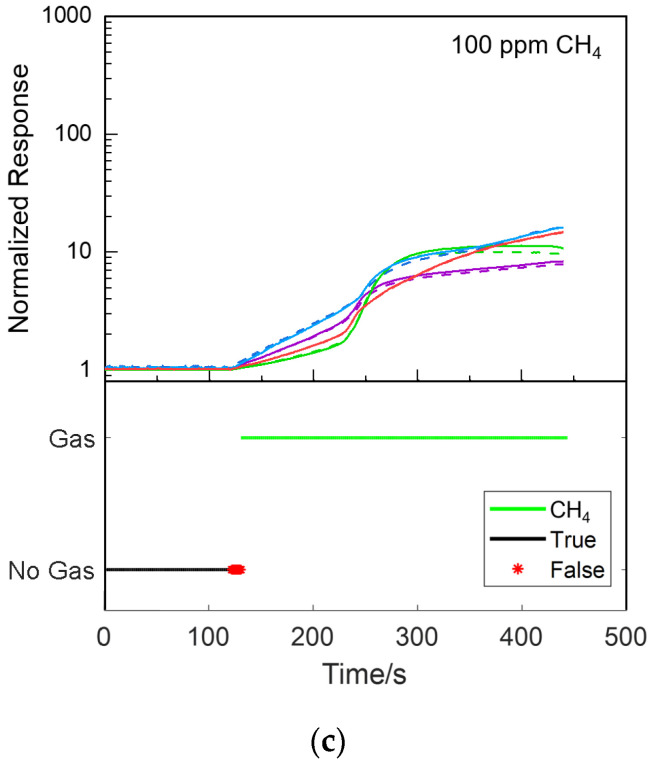
External validation results of CLSTM neural network model using repeated sensor array responses towards (**a**) 100 ppm CO, (**b**) 100 ppm C_3_H_8_ and (**c**) 100 ppm CH_4_. Note: solid lines are testing data and dash lines are training data.

To further demonstrate the capability of CLSTM neural network model for potential real-time predications, we conduct the external validation analysis by using one additional set of sensor array response data towards 100 ppm CO, 100 ppm C_3_H_8_ and 100 ppm CH_4_, respectively. Prior to the analysis, the CLSTM model is well re-trained offline upon all data utilized in preceding section, where 40 data points (2 s) are included in each time-series sample. The additional validation dataset is experimentally acquired at a different time, which is inevitably subject to the ambient and measurement variations. Such variations further result in the discrepancy between the training and validation datasets as shown in Figure 10. For this reason, these two datasets are statistically independent and thus can be readily used for the external validation analysis. The prediction results are listed in Table 4. As can be seen, only a very small number of misclassifications are captured, most of which occur right after the reducing gas introduction (Figure 10). Recall that each sample lasts 2 s with 0.5 overlapping ratio. The misclassification number (3rd column of Table 4) clearly illustrates that the model is capable of identifying gas species correctly starting from 13 s upon the exposure and throughout the entire transitional phase. It is not surprisingly found that the CLSTM appears to be a bit delay in response towards reducing gas exposure because it falsely identifies “No Gas” class at the initial stage of exposure. On the other hand, C_3_H_8_ becomes a more dominant interference than “No Gas” class in CO testing scenario. Overall, the external validation results demonstrate the excellent capability of CLSTM in differentiating/identifying different gas species at early stages. 

To further enhance the early-stage gas identification capacity to suit the practical real-time application, the fundamental study needs to be continuously conducted in order to create a sensor array with improved selectivity and sensitivity with respect to the target gases. Additionally, the efficient and effective signal processing technique can be incorporated to extract the pivot differentiable features from the raw data, which facilitates the neural network learning. It is also worth mentioning that, since in most practical situations the gases are mixed, accurate identification of gas concentrations bears practical significance. Generally, this can be fulfilled by resorting to the regression type of analysis, which often requires the response data in equilibrium. Predicting the gas concentrations of a gas mixture during the transitional state is still challenging. This will be subject to future research.

## 6. Conclusions

In this study, we develop a novel gas identification framework based on a sensor array for high-temperature applications that leverages both the experimental and numerical techniques. Sensing materials in the array are selected to ensure good sensitivity and diverse sensing characteristics towards gas analytes of interest. A machine learning concept then is proposed to realize the automatic gas identification during transitional state without manual interference based upon the recorded real-time sensor array data. To characterize the intrinsic temporal correlation within time-series data and improve the identification accuracy accordingly, the convolutional long short-term memory (CLSTM) neural network with designed architecture configuration is developed in this study. Its accuracy and robustness have been systematically investigated and compared with that of baseline models, i.e., MLP and SVM. The results illustrate that CLSTM yields higher than 95% classification accuracy in all proposed testing scenarios, and completely outperforms the baseline models. More importantly, it has the enhanced capability of gas identification at early stages, in which the sensor data features of different gases are slightly differentiable. This shows the promise of the proposed methodology for the future real-time applications.

## Figures and Tables

**Table 1:** Data labeling and curation for classification analysis.

CO (ppm)			CH_4_ (ppm)			C_3_H_8_ (ppm)			No Gas
50	80	100	50	80	100	50	80	100	
Class 1			Class 2			Class 3			Class 4
381 samples			381 samples			381 samples			378 samples

**Table 2:** Layer configuration of CLSTM neural network model.

Layer ID	Layer Type	Size	Output Shape	Parameters
#1	Input	1×25×4	1×25×4	0
#2	Convolutional (ReLU)	1×3×16	1×25×16	288
#3	Convolutional (ReLU)	1×3×16	1×25×16	784
#4	Max pooling	1×2	1×12×16	0
#5	Fully connected (Flatten)	No	192	0
#6	LSTM	100	100	117,200
#7	Fully connected (ReLU)	100	100	10,100
#8	Output (Softmax)	4	4	404

Note, the total number of trainable parameters in this model is 248,264.

**Table 3:** Comparison of models’ overall prediction performance.

Models	Data Points Per Sample	Accuracy	Standard Deviation
CLSTM	100	95.79%	1.98%
MLP	100	87.82%	4.48%
SVM	100	77.75%	3.23%
CLSTM	40	96.76%	2.67%
MLP	40	91.22%	2.74%
SVM	40	79.02%	3.02%

**Table 4:** External validation results.

Gas	Accuracy	# of Misclassifications (442 Samples in Total)
100 ppm CO	97.06%	13
100 ppm C_3_H_8_	97.28%	12
100 ppm CH_4_	97.96%	9

## Data Availability

Not applicable.
